# Breast cancer histopathological image classification based on collaborative multi-domain feature learning

**DOI:** 10.1371/journal.pone.0341320

**Published:** 2026-01-27

**Authors:** Lingfei He, Hongping Hu, Rong Cheng

**Affiliations:** School of Mathematics, North University of China, Taiyuan, Shanxi, China; Bayer Crop Science United States: Bayer CropScience LP, UNITED STATES OF AMERICA

## Abstract

Breast cancer is a highly heterogeneous malignant tumor, and its accurate classification is of great significance for clinical diagnosis and treatment decision-making. In recent years, convolutional neural networks and Transformer have been widely used in pathological image analysis of breast cancer. Though the former excels at capturing local information and the latter is adept at modeling global dependencies, the former is limited by fixed sampling positions and is hindered to characterize irregular cell morphology, the latter is insufficient in describing the two-dimensional spatial structure of cells and tissues. Based on these, a Spatial–Frequency Domain Feature Extraction Model (S-FDFEM) proposed in this paper integrates spatial and frequency domain information to enhance feature learning for pathological image recognition. Specifically, in the spatial domain, Deformable Bottleneck Convolution (DBottConv) is utilized to effectively represent intricate cell and tissue morphological variations in pathological images and improve the expression ability of local features; in the frequency domain, wavelet low frequencies and Fourier high frequencies are generated based on the input pathological images to capture global approximation and fine structures, then statistical transformer and depth gradient feature extraction modules are integrated to operate on these two frequency domain components, enabling global dynamic focusing and two dimensions spatial characterization of pathological images. Experimental results show that the classification of breast cancer pathological image on BreakHis and BACH datasets verify the superiority of the S-FDFEM proposed in this paper.

## 1 Introduction

Breast cancer remains the most prevalent malignancy among women globally, representing a major contributor to cancer-related morbidity and mortality [1–4]. The increasing incidence of this disease, driven by complex interactions among genetic, hormonal, environmental, and lifestyle factors, underscores the urgent need for improved diagnostic and therapeutic strategies. Early and accurate detection is crucial for enhancing patient outcomes, reducing mortality rates, and enabling personalized treatment approaches. While traditional diagnostic methods such as mammography, ultrasonography, and tissue biopsy have advanced clinical practice, they still present significant limitations. Mammography, the primary screening tool, has reduced sensitivity in dense breast tissue, particularly in younger women, which can result in false-negative findings [5]. Although ultrasonography is useful for differentiating benign from malignant lesions, it lacks the spatial resolution necessary to detect small or early-stage tumors [6]. Tissue biopsy, although definitive, is invasive, costly, and impractical for routine screening [7], highlighting the need for robust, non-invasive, and accurate diagnostic methods.

Histopathological analysis, enabled by Whole Slide Imaging (WSI), has become the gold standard for breast cancer diagnosis, as it provides detailed insights into cellular morphology, tissue architecture, and molecular marker profiles [8]. However, manual histopathological evaluation is time-consuming, prone to inter-observer variability, and limited by human cognitive capacity [9].

In recent years, advancements in deep learning technologies, specifically convolutional neural networks (CNNs) and transformers, have greatly improved the extraction of features from pathological images. These methods, known for their end-to-end learning capability, robust nonlinear modeling performance, and effective representation of intricate semantic relationships within images, have demonstrated outstanding performance in analyzing high-dimensional pathological image data. As a result, they have effectively addressed critical challenges in pathological image classification [10]. CNNs have demonstrated considerable success in extracting local features from pathological images. For instance, Xu et al. [11] developed a multidimensional feature extraction network MDFF-NET which integrated both one-dimensional and two-dimensional convolutional operations to effectively capture intricate pathological characteristics and encode high-level semantic information from histopathological image data. George et al. [12] detected non-overlapping cell nucleus blocks from histopathology images and proposed a low-complexity CNN for feature extraction. The extracted CNN features were then classified using a strategy that combined feature fusion with a support vector machine (FF+SVM). LI.Xia et al. [13] proposed a novel breast cancer histopathological image classification network (BC-MBINet) based on multiple convolutional layers, achieving an accuracy of 99.04%. Joseph et al. [14] proposed a fully automated and robust dual multi-scale convolutional neural network capable of extracting linearly separable and scale-invariant features to address the challenges posed by variations in resolution, texture diversity, and high coherence in breast cancer histopathological images. Traditional convolution operations are constrained by a fixed receptive field, limiting their ability to capture the global structural information of lesion regions and thereby hindering further improvements in classification performance. Transformers address this limitation by effectively modeling global context and long-range dependencies. Li et al. [15] incorporated BIFormer into the feature extraction stage to strengthen global feature interactions and improve semantic information transfer in histopathological images. Similarly, Hao et al. [16] proposed ST Double Net, a two-stage Swin Transformer–based architecture that integrates global and local features to enhance feature diversity. Gao et al. [17] further introduced HTransMIL, a Transformer-based hybrid multiple instance learning framework that captured comprehensive contextual information and improves inter-class discriminability.

Therefore, CNNs and Transformer extract features from local and global areas, respectively, and finally the high-resolution image is transformed into a set of high-dimensional features containing rich information. Although they have achieved good results in the classification of breast cancer pathological images, they still have some limitations, limiting the full representation of pathological features. On the one hand, CNNs rely on fixed size convolutional kernels to extract features within the local receptive field. This static sampling method is difficult to adaptively model irregular tissues and cell morphology in complex pathological images, resulting in shortcomings in characterizing the microstructure and overall morphology of the model. On the other hand, through its self-attention mechanism, Transformer can automatically model the long-range dependencies between any pixel region in the image, effectively promoting the interaction of cross regional tissue structure and cell morphology information in pathological images. However, the self-attention mechanism has limitations in processing pathological images: it lacks explicit modeling ability for two-dimensional spatial structures, making it difficult to accurately perceive the arrangement and spatial distribution patterns of cell populations. And these morphological spatial features are precisely important pathological basis for distinguishing cancer subtypes and evaluating malignancy [18].

To this end, we propose a spatial-frequency domain feature extraction model (S-FDFEM) that fully considers irregular cell and tissue morphology in the spatial domain, and introduces frequency domain characteristics to promote the two-dimensional spatial information expression of pathological images. The main contributions can be summarized as follows:

1. Design a novel multi-domain collaborative feature learning architecture, Spatial–Frequency Domain Feature Extraction Model (S-FDFEM), which can combine local, global and spatial representations to enhances the ability to capture complex, multi-morphological structures in pathological images.

2. We propose a frequency domain global-spatial feature extraction module (FD-GSFE), which is used to enhance the perception of global and two-dimensional spatial features from breast cancer pathological images, so that the model can learn features that are critical to cancer discrimination.

3. We design a spatial domain feature extraction (SDFE) module that adaptively captures local features of irregular tissue morphology in cancer regions, effectively overcoming the limitations of fixed receptive fields in traditional convolutional kernels and further improving feature representation and classification performance.

## 2 Related work

**Convolutional neural networks (CNNs):** CNNs have become a cornerstone in image analysis.Classical architectures such as ResNet, GhostNet, MobileNet, and ShuffleNet have demonstrated strong performance by effectively capturing local morphological features, including nuclear shape and tissue structure, thereby achieving high classification accuracy [19–22]. To further enhance feature representation, Zou et al. [23] proposed DsHoNet, which combines covariance pooling with Ghost modules to construct a lightweight network enriched with high-order features. Liu et al. [24] developed CTransNet, leveraging pretrained DenseNet and transferred learning to achieve strong cross-dataset generalization. Hou et al. [25] introduced a method integrating convolutional Long Short Term Memory(LSTM) with an adaptive weighted bilateral multidimensional attention mechanism, improving feature quality while mitigating class imbalance. However, these approaches rely on convolution kernels with fixed receptive fields. When applied to structurally complex and morphologically diverse breast cancer histopathological images, their local receptive fields are limited, restricting the ability to adapt to irregular tissue distributions and cellular arrangements. To address this limitation, we propose a Deformable Bottleneck Convolution (DBottConv) structure that dynamically adjusts convolution sampling locations through learnable offsets, enabling flexible extraction of discriminative pathological features.

**Histogram of Oriented Gradients (HOG):** HOG plays a crucial role in image feature representation, effectively capturing the gradient magnitude and direction of each pixel [26]. This method has been widely applied. For example, R. Newlin Shebiah et al. [27] combined HOG with cascaded AdaBoost classifiers for human body part detection, achieving promising results, while Yang et al. [28] improved HOG and applied it to spectral image recognition, enabling polarization spectral analysis. However, when processing structurally complex breast cancer histopathological images, traditional HOG relies solely on horizontal and vertical gradient calculations, limiting its ability to capture irregular pathological features. To address this limitation, we propose a depth gradient feature extraction module that extends gradient computation to diagonal directions to enhance multi-directional structural perception. Finally, we integrated it into the Fourier high-frequency spatial feature extraction branch to enhance two-dimensional spatial perception.

**Fig 1:**
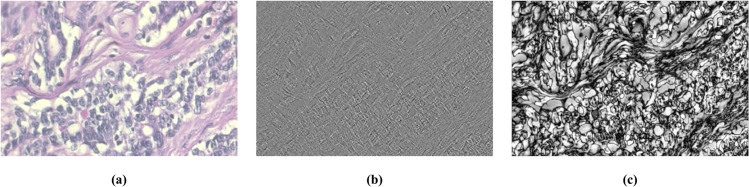
(a) primary breast cancer pathological image (b) high frequency component of primary image from wavelet transform (c) high frequency component of primary image from Fourier transform.

**Frequency domain analysis:** The method provides a new methodological perspective for image processing, significantly enhances the expression ability of image features, and shows unique advantages in pathological image processing of breast cancer. In pathological images, high-frequency information usually corresponds to detailed features such as cell edges, texture structures, and dense area contours, which can enhance the recognizability of small shapes; Low frequency information reflects the global structure of the image, revealing the overall distribution characteristics of tissues and cells. For example, Li et al. [29] proposed a dual branch adaptive fusion network, which uses Fourier transform to enhance high-frequency and low-frequency components at the local and global feature levels respectively. By highlighting the complementarity between details and the overall structure, it improves the richness and discrimination of feature representation of breast cancer pathological images. Yan et al. [30] proposed DWNAT Net, which extracts deep frequency domain features based on discrete wavelet transform, further enhancing the frequency domain representation ability of pathological images and effectively improving classification performance. However, wavelet high frequency components contain significant noise, which distorts feature distribution and obscures key pathological details. The research by Tan J [31] shows that Fourier high frequency images have clear texture expression, highlighting edge and detail features (as shown in Fig 1). Therefore, we propose the Frequency Domain Global-Spatial Feature Extraction (FD-GSFE) module, which combines wavelet low-frequency and Fourier high-frequency representations to simultaneously capture global structural approximations and fine pathological details, thereby improving the robustness and accuracy of feature representation.

**Spatial distribution characteristics:** Spatial distribution characteristics are crucial for cancer type discrimination in pathological images. At present, Graph Neural Network (GNN) is the most commonly used spatial feature extraction method, which aggregates and updates information between nodes by constructing the topological structure between image nodes, and has achieved good results in pathological image analysis. For example, Liu et al. [32] proposed a hierarchical pyramid model that integrates GNN into a pyramid structure to learn the geometric spatial representation of pathological images. However, when tissues and cells are densely arranged in pathological images, traditional graph neural networks are difficult to distinguish between cell and tissue boundaries, which can easily lead to misjudgment of the lesion area. To address this limitation, we introduce a Fourier high-frequency spatial feature extraction branch in the FD-GSFE module, which derives depth gradient features from high-frequency components to enhance the model’s two-dimensional spatial perception.

In summary, S-FDFEM addresses the limitations of prior models by introducing a space-frequency collaborative feature extraction framework that jointly leverages local, global, and spatial distribution information. Different from hybrid models such as DWNAT-Net [30], which primarily enhance feature representations within a single domain, S-FDFEM explicitly integrates complementary information across the spatial and frequency domains, fully leveraging the unique advantages of each domain to capture fine-grained features structural patterns. At the same time, the S-FDFEM combines frequency domain with two-dimensional spatial distribution for the first time, making up for the lack of spatial distribution characteristics of pathological images in existing methods.

## 3 Key theories and technologies

This section elaborates the theoretical methods used in this paper: wavelet transform, Fourier transform and statistical attention mechanism [33], in which the attention mechanism will be combined with the transformer architecture in the Sect 4.2.2 to form the Statistical Transformer.

### 3.1 Wavelet transform

High-frequency and low-frequency components are extracted through two-dimensional discrete wavelet transform in this study. Let $I\in R^{H\times W\times C}$ denote the input feature map. Using Haar wavelet transform to divide an image into four frequency domain sub bands, where the filtering used is shown in Eq (1):

$$L=\frac{1}{\sqrt{2}}{\left[1,1\right]}^{T},H=\frac{1}{\sqrt{2}}{\left[1,-1\right]}^{T},$$ (1)

$$I_{LL},[I_{LH},I_{HL},I_{HH}]=2D-DWT(I),$$ (2)

where, $I_{LL}\in R^{\frac{H}{2}\times \frac{W}{2}\times C}$ represents the low-frequency and $I_{LH},I_{HL},I_{HH}\in R^{\frac{H}{2}\times \frac{W}{2}\times C}$ represents the high-frequency components. Finally, the original image is reconstructed through the inverse wavelet transform (IWT) as follows:

$$I_{output}=2D-IWT(I_{LL},I_{LH},I_{HL},I_{HH}),$$ (3)

where, *I*_*output*_ effectively synthesizing the decomposed frequency components back into the spatial domain.

### 3.2 Fourier transform

Assuming the input image is $X\in R^{H\times W\times C}$. The input image undergoes frequency transformation via the fast Fourier transform (FFT), transitioning from spatial to frequency domain representation. The mathematical formulation of this conversion is expressed as:

$$F(m,n)\;=\frac{1}{HW}\sum_{h=0}^{H-1}\sum_{w=0}^{W-1}X(i,j)e^{-j2\pi (\frac{hm}{H}+\frac{wn}{W})}.$$ (4)

To achieve frequency-selective decomposition, frequency separation is performed through Gaussian filtering in the Fourier domain. This process involves:

$$H_{i}(m,n)=\left\{ \begin{array}{cc} 1-e^{-\frac{D(m,n{)}^{2}}{2D_{0}^{2}}}, & D(m,n)=\sqrt{(m-M/2{)}^{2}+(n-N/2{)}^{2}},\\ {}[6pt]e^{-\frac{D(m,n{)}^{2}}{2D_{0}^{2}}}, \end{array}\right.$$ (5)

where, i∈(high,low), *D*(*m*,*n*) and *D*_0_ are cut-off frequencies. Multiply the frequency domain with the filter to obtain the filtered high-frequency component *G*_*high*_(*m*,*n*) and low-frequency component *G*_*low*_(*m*,*n*).

Finally, due to the fact that high-frequency features are only utilized, the inverse Fourier transform is applied to the high-frequency component, reconstructing a spatial-domain image:

$$X_{\text{high}}(x,y)=\frac{1}{HW}\sum_{h=0}^{H-1}\sum_{w=0}^{W-1}G_{\text{high}}(u,v)\;e^{j2\pi \left(\frac{hm}{H}+\frac{wn}{W}\right)}.$$ (6)

### 3.3 Statistical attention

Let the input image be denoted as $I\in R^{B\times H\times W\times C}$, where H×W is the spatial dimension and *C* is the number of channels. The specific steps of statistical attention [33] are as follows:

Firstly, a linear transformation followed by a rearrangement operation is applied to *I* to generate multi head representation:

$$X=T(WI)\in R^{B\times h\times N\times d},$$ (7)

where, *W* is the projection matrix and *T* is the rearrangement operation. $X\in {\mathbb{R}}^{B\times h\times N\times d}$, N=H×W, *d* = *C*/*h*, *d* represents the dimension of each head and *h* represents the number of attention heads. Subsequently, normalize *X* along the *N* dimension according to the $L_{2}$ norm to obtain *O* and the attention distribution *A*:

$$A=Softmax(\sum_{d}O\cdot \tau )\in R^{B\times h\times N},O=\frac{X-\mu }{\sqrt{\sigma^{2}+\varepsilon }}⬝\gamma +\beta .$$ (8)

where, μ and $\sigma^{2}$ are the mean and variance of the *N* dimension, *γ* and *β* are learnable parameters, and ε is a small constant to prevent the denominator from being zero. *τ* is a learnable temperature parameter used to scale the attention distribution.

Then, the attention scores are computed:

$$A_{norm}=\frac{A}{\sum_{N}A+\varepsilon }\in {\mathbb{R}}^{B\times h\times N},\;A_{norm}=unsqueeze(A_{norm})\in {\mathbb{R}}^{B\times h\times 1\times d},\;dots=(A_{norm}\cdot O^{2})\in {\mathbb{R}}^{B\times h\times 1\times d},$$ (9)

where, *dots* denotes the attention score, *A*_*norm*_ represents the normalized value of *A*, *unsqueeze* represents adding a new dimension to the third dimension.

Next, the weighted image is obtained by multiplying the attention weight with the attention weight *attn* and matrix *O*:

$$output_{weigh}=-O\cdot (A\cdot attn)\in R^{B\times h\times N\times d},attn=\frac{1}{1+dots}\in R^{B\times h\times 1\times d}.$$ (10)

Finally, The weighted image *output*_*weigh*_ is processed through a LN layer and a MLP layer to get the output features, and the output features is rearranged to B×N×C size, and the final restored image size is $X\in R^{B\times H\times W\times C}$.

## 4 Proposed model

### 4.1 Overall architecture

**Fig 2:**
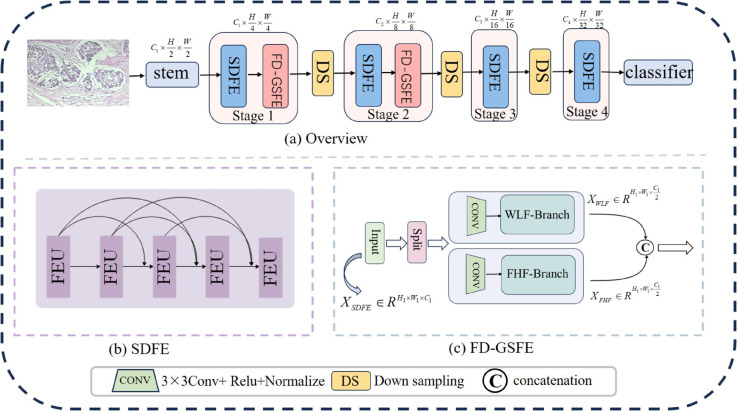
S-FDFEM model. (a) Structure diagram of the S-FDFEM model. (b) Composition structure of the SDFE module. (c) Composition structure of the FD-GSFE module.

We propose a spatial-frequency domain feature extraction model (S-FDFEM). As illustrated in Fig 2, S-FDFEM is designed based on a pyramid structure, which can extract multi-scale fine-grained features. It consists of a stem and four stages. The stem performs preliminary processing on pathological images, and the subsequent four stages are used to extract shallow and deep pathological features. Due to the rich and relatively complete pathological information contained in shallow features, we only apply Frequency Domain Global-Spatial Feature Extraction (FD-GSFE) to the first two stages of the pyramid structure, and the output of the SDFE module is directly used as the input of the FD-GSFE module. Finally, the classifier outputs its category. Specifically, we use Spatial Domain Feature Extraction (SDFE) as the backbone to capture local features. FD-GSFE captures global and spatial perception.

Next,we explore these main modules separately.

### 4.2 Composition of S-FDFEM model

#### 4.2.1 SDFE module.

**Fig 3:**
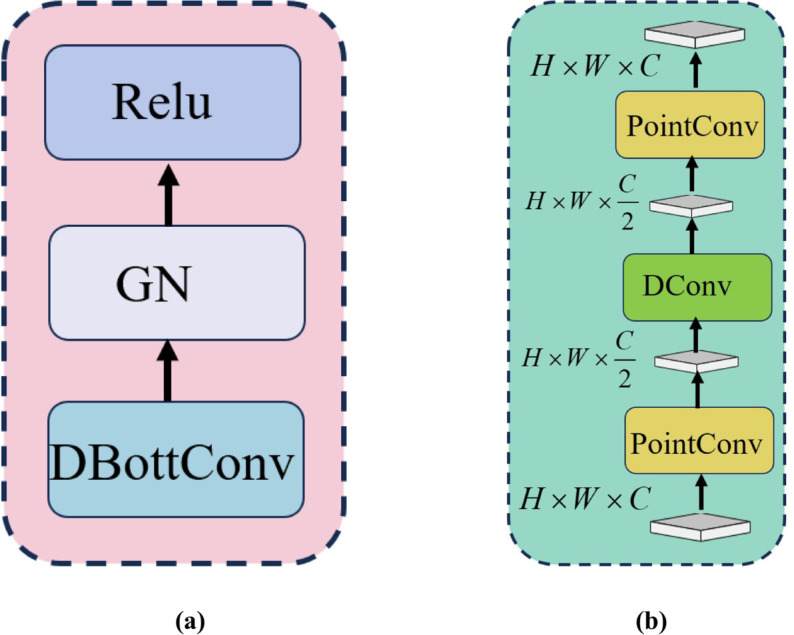
Two compositions of SDFE module (a) FEU structure (b) DBottConv structure.

Deformable Convolution parameterizes sampling positions through learned offsets, enabling flexible receptive field adaptation [34] and better alignment with irregular morphological features in tumor regions; Bottleneck convolution (BC) compresses feature representations into a compact low-dimensional form, thereby reducing computational complexity and enhancing efficiency while preserving representational capacity [35]. Therefore, we integrate deformable and bottleneck convolutions to devise the Deformable Bottleneck Convolution (DBottConv), which effectively captures local features from irregular cellular and tissue structures. Specifically, a DBottConv, Group Normalization (GN), and Relu form a Feature Enhancement Unit (FEU). Based on this, we designed a SDFE module, as shown in Fig 2(b), which is densely connected by multiple feature enhancement units. Fig 3 gives two important compositions of SDFE module.

In the SDFE module, each Feature Enhancement Unit (FEU) first derives local features with adaptive receptive fields using Deformable Bottleneck Convolutions (DBottconv), which begins with a pointwise convolution for channel compression to reduce computational overhead, followed by a deformable convolution to capture irregular morphological patterns in lesion regions, and concludes with another pointwise convolution to restore the original channel dimensions. Subsequently, group normalization and an activation function are applied to enhance training stability and convergence. Finally, the outputs from *N* FEUs are aggregated via dense connections to form the module’s final output of SDFE module.

#### 4.2.2 FD-GSFE module.

Global feature extraction via Transformer-based methods plays a pivotal role in breast cancer histopathological image classification. However, Transformers often lack inherent two-dimensional spatial awareness. To address this, we propose the FD-GSFE module, which employs a dual-branch architecture (Fig 2(c)). This framework comprises two complementary components: the Wavelet Low-Frequency (WLF) branch for global feature extraction and the Fourier High-Frequency (FHF) branch for spatial feature extraction. Together, these branches enable cooperative global dynamic focusing and enhanced 2D spatial perception, fostering robust pathological image analysis.

Suppose the output features generated by the SDFE module to be $X_{SDFE}\in R^{H_{1}\times W_{1}\times C_{1}}$. To save computing resources, the input features are segmented on the channel and used as inputs for two branches $X_{WLF}\in R^{H_{1}\times W_{1}\times \frac{C_{1}}{2}}$ and $X_{FHF}\in R^{H_{1}\times W_{1}\times \frac{C_{1}}{2}}$. *X*_*WLF*_ and *X*_*FHF*_ are fed into the WLF branch and FHF branch through a 3×3 convolutional layer. Finally, the outputs of the two branches are concatenated at the channel level and represented as $X_{FD-GSFE}\in R^{H_{1}\times W_{1}\times C_{1}}$.

**A. WLF Branch:**

**Fig 4:**
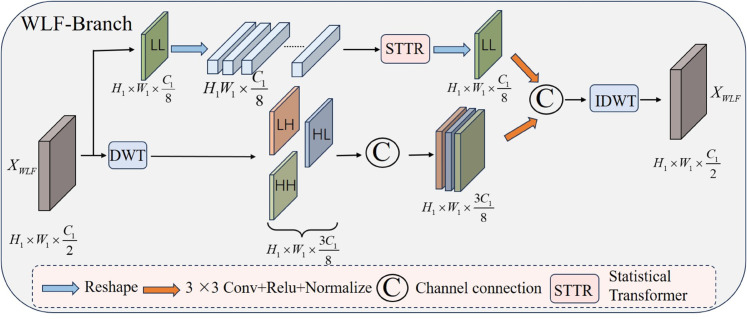
Structure of WLF-Branch.

As show in Fig 4, For the input data *X*_*WLF*_, we utilize wavelet transform to perform frequency domain decomposition:

$$I_{LL},[I_{LH},I_{HL},I_{HH}]=DWT(X_{WLF}),$$ (11)

where, *DWT* represents wavelet transform, $I_{LL}\in R^{H_{1}\times W_{1}\times \frac{C_{1}}{8}}$ represents low-frequency component, and $[I_{LH},I_{HL},I_{HH}]\in R^{H_{1}\times W_{1}\times \frac{3C_{1}}{8}}$ represents high-frequency component. Subsequently, global features are extracted using low-frequency components, while high-frequency components enhance lesion features:

$$X_{Glob\text{al}}=STTR(I_{LL}),$$ (12)

$$X_{En}=Conv([I_{LH},I_{HL},I_{HH}]),$$ (13)

where, *STTR* represents the Statistical Transformer and $X_{Glob\text{al}}\in R^{H_{1}\times W_{1}\times \frac{C_{1}}{8}}$ is the pathological feature after global dynamic focusing, which realizes the interaction and information exchange between pixels. *Conv* is composed of 3×3 convolution, Relu activation and normalization to enhance lesion features. Finally, the low-frequency and high-frequency images are concatenated on the channel, and the images are restored to the spatial domain using inverse wavelet transform:

$$X_{WLF}=IDWT[C\text{oncat}(I_{LL},I_{LH},I_{HL},I_{HH})],$$ (14)

where, *IDWT* is the inverse wavelet transform, and Concat represents the concatenation of high-frequency and low-frequency features on the channel.

**Fig 5:**
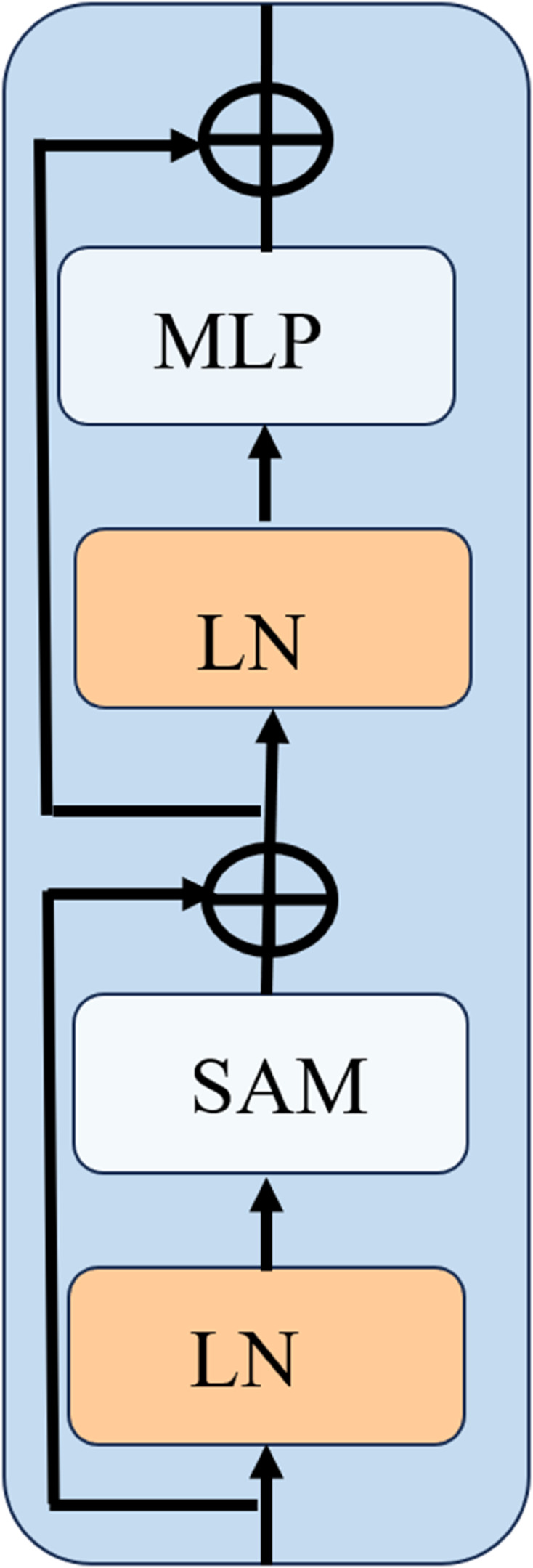
Chart of statistical transformer.

**Statistical Transformer:** The traditional Transformer architecture consists of Layer Normalization (LN), Multi-Head Self-Attention (MSA), a Multi-Layer Perceptron (MLP), and Residual Connections (RES). However, MSA models only first-order feature similarities through pixel-wise dot products, limiting its ability to capture higher-order statistical dependencies. To address this, we introduce a Statistical Attention Mechanism (SAM) that constructs data-adaptive low-rank projections based on the empirical second-order moments of pixel features, enhancing high-order feature modeling [33]. Replacing MSA with SAM yields the Statistical Transformer, which effectively captures second-order statistics and deepens feature representations. Fig 5 illustrates the detailed process, while its mathematical formulation is expressed as follows.

The input features $F\in R^{H\times W\times C}$ undergo initial Layer Normalization (LN), followed by processing through the Statistical Attention Mechanism (SAM) to capture global contextual interactions and produce an enhanced representation. This enhanced representation is then fused with the original input *F* via a pixel-wise residual connection, yielding an intermediate output.

$$F_{1}=SAM(LN(F))+F.$$ (15)

Subsequently, the intermediate output *F*_1_ is subjected to another LN layer and a Multi-Layer Perceptron (MLP), with the result added to the intermediate *F*_1_ via a residual connection to generate the final output features of the Statistical Transformer.

$$F_{2}=MLP(LN(F_{1}))+F_{1},$$ (16)

where, *F*_2_ represents the output features of the Statistical Transformer.

**B.FHF-Branch:**

**Fig 6:**
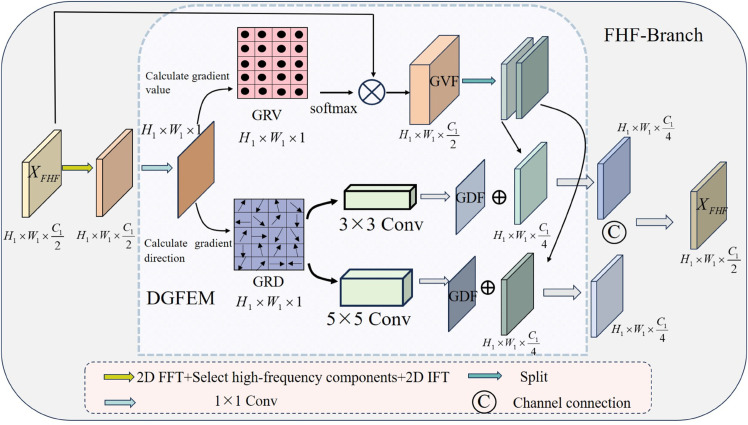
Structure of FHF-Branch.

In breast cancer histopathological images, Fourier high-frequency enhancement is employed to accentuate fine structural details. Subsequently, a Depth Gradient Feature Extraction Module (DGFEM) is applied to these enhanced contours to capture intricate two-dimensional spatial features. Fig 6 illustrates the architecture of the FHF-Branch, where DGFEM forms the core component.

Assume the input image is denoted as $X_{FHF}\in R^{H_{1}\times W_{1}\times \frac{C_{1}}{2}}$. First, the high-frequency image is derived via Fourier transform:

$$X_{\text{high}}=FFT(X_{FHF}),$$ (17)

where, *FFT* represent the Fourier transforms, $X_{\text{high}}\in R^{H_{1}\times W_{1}\times \frac{C_{1}}{2}}$, A 1×1 convolution then compresses the channels of $X_{\text{high}}$ to 1, yielding the feature map $X_{\text{high}}\in R^{H_{1}\times W_{1}\times 1}$, which reduces computational overhead.

The Gradient Value Feature (GVF) and Gradient Direction Feature (GDF) are computed as:

$$X_{GVF},X_{GDF}=DGFEM(X_{\text{high}}),$$ (18)

where, DGFEM represents depth gradient feature extraction modules, $X_{GVF}\in R^{H_{1}\times W_{1}\times \frac{C_{1}}{2}}$, $X_{GDF}\in R^{H_{1}\times W_{1}\times 2}$. The GVF emphasizes pathological cell and tissue edges, while the GDF captures the geometric spatial arrangement of boundaries across varying receptive fields, consisting of *X*_*S*_ and *X*_*B*_:

$$X_{S},X_{B}=Split(X_{GDF}),$$ (19)

where, Split represents dividing the channel, $X_{S},X_{B}\in R^{H_{1}\times W_{1}\times 1}$. Then, we split the GVF on the channel, adding them to the *X*_*S*_ and *X*_*B*_ at the pixel level respectively, representing the addition of their two-dimensional spatial information to each pixel point:

$$X_{1},X_{2}=S\text{plit}(X_{GVF}),$$ (20)

$$X_{S1}=X_{S}+X_{1},\;X_{B2}=X_{B}+X_{2},$$ (21)

where, *X*_*S*1_ and *X*_*B*2_ represent learning two-dimensional spatial features of different scales, and finally concatenating these two features at the channel level:

$$X_{FHF}=Concat(X_{S1},X_{B2}).$$ (22)

**DGFEM Module:** Traditional Histogram of Oriented Gradients (HOG) [26] highlights edge magnitudes via gradient values and reflects angular variations in gradient directions. However, it often overlooks diagonal responses (e.g., at $45^{\circ }$ and $135^{\circ }$), leading to inadequate representation of subtle morphological changes in complex pathological images.To mitigate this, the DGFEM module extends HOG by incorporating diagonal gradient values ($45^{\circ }$, $135^{\circ }$) and refining direction calculations. Gradient values are enhanced via softmax to emphasize edges, while directions capture geometric arrangements of cell and tissue boundaries. Dual convolutions with varying kernel sizes expand the receptive fields for spatial learning.

Assume the feature map *F* is H×W×1. Four directional operators compute gradients:

$$\nabla_{x}=[-1,\;0,\;1],\;\nabla_{y}=[\begin{array}{c} -1\\ 0\\ 1 \end{array}],\;\nabla_{45^{\circ }}=[\begin{array}{cc} 0 & -1\\ 1 & 0 \end{array}],\;\nabla_{135^{\circ }}=[\begin{array}{cc} -1 & 0\\ 0 & 1 \end{array}].$$ (23)

The gradient value for each direction is $G_{d}=\nabla_{d}\;⊗\;F,d\in \{x,y,45^{\circ },135^{\circ }\}$, ⊗ represents the gradient value calculation operation for each direction. Subsequently, the gradient values of each pixel can be obtained:

$$g=\sqrt{{G_{x}}^{2}+{G_{y}}^{2}+{G_{45^{\circ }}}^{2}+{G_{135^{\circ }}}^{2}}\in R^{H\times W\times 1}.$$ (24)

Edge enhancement yields the Gradient Value Feature (GVF) via softmax:

$$g_{soft\max}=Soft\max(g),$$ (25)

where, $g_{soft\max}\in R^{H\times W\times 1}$, project the gradient value in the diagonal direction onto the horizontal and vertical directions:

$$g_{x}^{total}=g_{x}+\frac{\sqrt{2}}{2}g_{45^{\circ }}+\frac{\sqrt{2}}{2}g_{135^{\circ }},\;g_{y}^{total}=g_{y}+\frac{\sqrt{2}}{2}g_{45^{\circ }}+\frac{\sqrt{2}}{2}g_{135^{\circ }}.$$ (26)

Finally, the gradient direction (GRD) *θ* is as follows:

$$\theta =\arctan\frac{g_{x}^{total}}{g_{y}^{total}}.$$ (27)

The GRD forms the gradient direction matrix of the feature map. To derive deep spatial feature distributions, a small-kernel convolution (SConv, 3×3) and a large-kernel convolution (BConv, 5×5) are applied to this matrix, promoting multi-scale spatial learning. The resulting distributions are concatenated channel-wise and normalized to yield the Gradient Direction Feature (GDF) it’s scale is H×W×2.

## 5 Experiments

### 5.1 Dataset

To ensure a comprehensive evaluation, we conduct experiments using both the BreakHis and BACH datasets. The BreakHis dataset contains 7,909 breast tumor histopathology images obtained under a microscope at four magnification levels (40×, 100×, 200×, and 400×). It includes both benign and malignant cases, which are further classified into eight subtypes based on pathological characteristics (Table 1). Depending on the task configuration, this dataset can be used for binary (benign vs. malignant) classification or eight-class classification. The BACH (Breast Cancer Histology) dataset contains 400 pathological images, categorized into four classes with 100 images each (Table 1). All images in both datasets were manually annotated by experienced pathologists to ensure high annotation reliability. It is worth noting that the BreakHis dataset exhibits significant class imbalance in the eight-class classification setting, which may influence model performance. However, this work focuses primarily on the model design for breast histopathology image classification and does not address data imbalance.

**Table 1:** Dataset categories.

Dataset	Categories							
BreakHis	Benign				Malignant			
	Adenosis	Fibroadenoma	Lobular tumor	Tubular adenoma	Ductal carcinoma	Lobular carcinoma	Mucinous carcinoma	Papillary carcinoma
BACH	Normal tissue		Benign lesion		In Situ carcinoma		Invasive carcinoma	

### 5.2 Experimental setup

The S-FDFEM performs the binary classification and eight-class classification on the Breakhis dataset, and the four-class classification on the BACH dataset. Data augmentation techniques are applied to expand the BreakHis dataset, the specific augmentation methods are: rotation, translation, contrast enhancement, saturation enhancement; Referring to the processing method [36], we exclude plaques with a core count of less than 30 in BACH and regenerate data based on samples from each category. The parameters of S-FDFEM are shown in Table 2. The evaluation indicators used in this experiment are accuracy, precision, F1-score and recall. To ensure a fair comparison, all models in the experiments were implemented using the same parameter configurations as the proposed S-FDFEM.

**Table 2:** Model parameters.

Parameter	Configuration
Split ratio of training and testing sets	7 (training): 3 (testing)
Image size	224×224
Iterations	200
Batch size	32
Learning rate	3×10^−5^
GPU	NVIDIA RTX-3060
Loss function	Focal-loss function

### 5.3 Experimental results of S-FDFEM model

#### 5.3.1 Breakhis dataset.

Tables 3 and 4 are the results of the binary and 8-class classification on the BreakHis dataset by the S-FDFEM model, respectively. From Table 3, the classification accuracies reach 99.33%, 99.36%, 98.67%, and 98.53% at 40×, 100×, 200×, and 400× magnifications, respectively, and the recalls, precisions and F1-Scores areconsistently above 98%. From Table 4, the classification accuracies at 40×, 100×, 200×, and 400× magnification reach 94.11%, 92.91%, 91.17% and 89.87%, respectively, both recall and precision remain above 87%. Especially, the reduction of the amount of organization information under fixed clipping size and the serious imbalance of data (for example, the sample sizes in the 200× test set vary from 33 to 268) cause the classification performance of S-FDFEM to be declined.

**Table 3:** Binary classification performance of S-FDFEM model at each magnification in BreakHis dataset.

Magnification	Accuracy (%)	Precision (%)	Recall (%)	F1-Score (%)
40×	99.33	99.08	99.37	99.22
100×	99.36	98.98	99.54	99.25
200×	98.67	98.45	98.45	98.45
400×	98.53	98.19	98.47	98.33

**Table 4:** 8-class classification performance of S-FDFEM model at each magnification in BreakHis dataset.

Magnification	Accuracy (%)	Precision (%)	Recall (%)	F1-Score (%)
40×	94.11	93.43	93.96	93.64
100×	92.91	93.16	92.57	92.79
200×	91.17	91.02	88.55	89.70
400×	89.87	89.99	87.54	88.71

#### 5.3.2 BACH dataset.

The evaluation results on the BACH dataset further demonstrate the strong classification capability of the S-FDFEM model. From Table 5, the model achieves an accuracy of 99.35%, precision of 99.37%, recall of 99.24%, and an F1-score of 99.30%. These results confirm the model’s strong generalization ability on breast cancer histopathology images.

**Table 5:** 4-Class classification performance of S-FDFEM model in BACH dataset.

Accuracy (%)	Precision (%)	Recall (%)	F1-Score (%)
99.35	99.37	99.24	99.30

### 5.4 Comparison between SDFE module and other modules

To verify the effectiveness of the SDFE module, we remove the FD-GFSE module from the first and second stages and constructed a new model consisting of only four SDFE modules, written as SDFE module, which is compared and analyzed with ResNet34 [37], Inception [38], and MobileNet [39].

#### 5.4.1 Breakhis dataset.

Experimental results on Breakhis dataset are presented in Table 6. At 40× and 100× magnifications, the proposed SDFE model achieves noticeably higher accuracy, precision, recall, and F1-score compared to all the compared modules. Although the Inception module shows a slight advantage in accuracy (<0.5%) at 200× and 400× magnifications, the SDFE module still maintains optimal classification effect from the perspective of comprehensive performance indicators. This advantage is attributed to the ability of deformable bottleneck convolution module to accurately capture irregular pathological features.

**Table 6:** Comparison of binary classification experiment results of SDFE module on BreakHis Dataset.

Model	40×				100×			
	Accuracy (%)	Precision (%)	Recall (%)	F1-Score (%)	Accuracy (%)	Precision (%)	Recall (%)	F1-Score (%)
ResNet34	95.74	95.53	94.51	94.99	93.01	91.18	93.00	94.99
MobileNet	94.73	92.92	95.73	94.09	93.97	95.14	90.91	92.94
Inception	96.99	96.33	96.72	96.52	96.38	96.47	95.01	95.70
SDFE	97.82	97.53	97.39	97.46	97.44	96.65	97.43	97.02
Model	**200×**				**400×**			
	**Accuracy (%)**	**Precision (%)**	**Recall (%)**	**F1-Score (%)**	**Accuracy (%)**	**Precision (%)**	**Recall (%)**	**F1-Score (%)**
ResNet34	96.52	97.04	94.80	95.82	95.87	96.00	94.49	95.19
MobileNet	96.26	96.88	94.00	95.01	96.14	95.87	95.08	95.08
Inception	97.26	98.09	95.56	96.71	96.52	95.95	96.09	96.02
SDFE	97.01	96.77	96.20	96.48	96.51	96.50	95.49	95.97

#### 5.4.2 BACH dataset.

The evaluation results on the BACH dataset are summarized in Table 7. ResNet34 achieves the highest accuracy (98.97%), while the SDFE model demonstrates more stable performance across accuracy, precision, recall, and F1-score. This result shows that Resnet34 is the best in classification effect and can accurately distinguish different categories, while the SDFE outperformed it in metric stability. Compared with MobileNet and Inception, the SDFE model achieves consistently higher performance.

**Table 7:** Compared results in four-class classification task on BACH Dataset.

Model	Accuracy (%)	Precision (%)	Recall (%)	F1-Score (%)
ResNet34	98.97	98.83	99.09	98.95
MobileNet	86.45	85.13	85.85	85.41
Inception	97.03	96.77	97.00	96.87
SDFE	97.26	97.19	97.27	97.16

### 5.5 Ablation experiments

To validate the S-FDFEM model, we conducted ablation experiments on both BreakHis and BACH datasets. Table 8 shows the module configuration of the four models in the ablation experiments. The SDFE module is shared across all four models. Baseline, Model A, Model B, and Model C are obtained by selectively enabling the WLF and FHF branches, where Model C corresponds to the proposed S-FDFEM.

**Table 8:** Ablation experiment configuration.

Model	SDFE	WLF-Branch	FHF-Branch
Baseline	✓	×	×
ModelA	✓	✓	×
ModelB	✓	×	✓
ModelC	✓	✓	✓

#### 5.5.1 BreakHis dataset.

**Fig 7:**
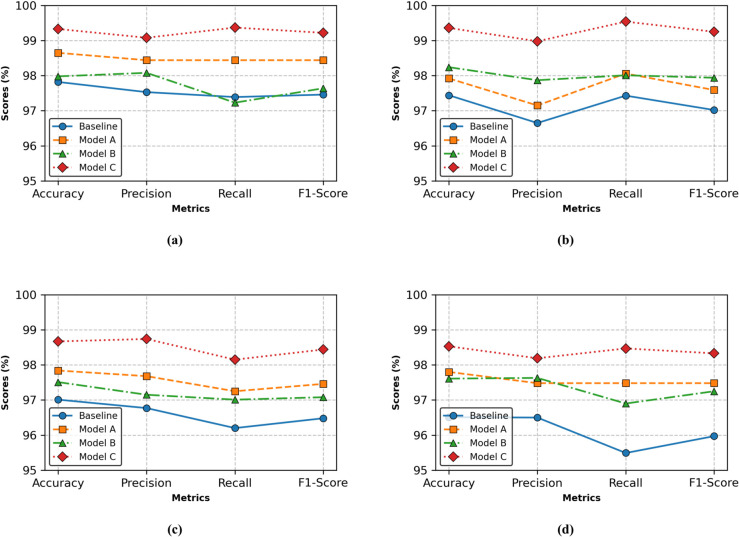
Visualization of the index of ablation experiments in the BreakHis dataset’s binary classification task.

Tables 9 and 10 present the binary and 8-class classification results on the BreakHis dataset for the Baseline, Model A, Model B, and Model C. Fig 7 shows the comparison curves of the four models on the binary classification task of the BreakHis dataset.

**Table 9:** Binary classification performance of ablation experiments on BreakHis Dataset.

Model	40×				100×			
	Acc (%)	Pre (%)	Rec (%)	F1-Sc (%)	Acc (%)	Pre (%)	Rec (%)	F1-Sc (%)
Baseline	97.82	97.53	97.39	97.46	97.44	96.65	97.43	97.02
ModelA	98.65	98.44	98.44	98.44	97.92	97.15	98.06	97.59
ModelB	97.98	98.08	97.23	97.64	98.24	97.87	98.01	97.94
ModelC	99.33	99.08	99.37	99.22	99.36	98.98	99.54	99.25
**Model**	**200×**				**400×**			
	**Acc (%)**	**Pre (%)**	**Rec (%)**	**F1-Sc (%)**	**Acc (%)**	**Pre (%)**	**Rec (%)**	**F1-Sc (%)**
Baseline	97.01	96.77	96.20	96.48	96.51	96.50	95.49	95.97
ModelA	97.84	97.68	97.25	97.46	97.80	97.48	97.48	97.48
ModelB	97.51	97.15	97.01	97.08	97.61	97.63	96.90	97.25
ModelC	98.67	98.74	98.15	98.44	98.53	98.19	98.47	98.33

**Table 10:** 8-class classification performance of ablation experiments on BreakHis Dataset.

Model	40×				100×			
	Acc (%)	Pre (%)	Rec (%)	F1-Sc (%)	Acc (%)	Pre (%)	Rec (%)	F1-Sc (%)
Baseline	91.41	90.97	90.02	90.13	90.66	89.97	89.12	89.34
ModelA	92.09	93.71	92.69	93.11	91.30	90.23	90.62	90.37
ModelB	92.59	91.10	91.62	91.26	91.95	91.14	90.81	90.92
ModelC	94.11	93.43	93.96	93.64	92.91	93.16	92.57	92.79
**Model**	**200×**				**400×**			
	**Acc (%)**	**Pre (%)**	**Rec (%)**	**F1-Sc (%)**	**Acc (%)**	**Pre (%)**	**Rec (%)**	**F1-Sc (%)**
Baseline	89.33	87.86	85.96	86.68	86.93	87.96	85.88	86.57
ModelA	90.50	90.00	88.00	88.00	88.40	88.71	86.92	87.60
ModelB	89.83	88.26	86.90	87.38	87.95	88.66	86.27	87.24
ModelC	91.17	91.02	88.55	89.70	89.87	89.99	87.54	88.71

When the magnification is 40×, 100×, 200×, and 400×, the binary classification accuracy of Baseline model on the Breakhis Dataset is 97.82%, 97.44%, 97.07% and 96.51%, respectively. The accuracy of eight-class classification is 91.41%, 90.66%, 89.33%, 86.93%, respectively. These results indicate that the use of deformable convolution causes the extraction of local irregular features of pathological images, improving local feature representation.

The WLF-branch leverages a statistical transformer to extract low-frequency global information in the wavelet frequency domain. With this branch added, Model A outperforms the Baseline by 0.48–1.29% in binary classification and 0.64–1.47% in eight-class classification, indicating enhanced global discrimination capability.

The FHF-branch utilizes the Fourier high-frequency component together with the DGFEM module to capture the two-dimensional spatial distribution of pathological structures. With this branch added, Model B improves binary classification accuracy by 0.16–1.10% and eight-class accuracy by 0.50–1.29%, demonstrating strengthened spatial feature representation.

Model C, which incorporates both the WLF-branch and FHF-branch, corresponds to the proposed S-FDFEM. It achieves the largest performance gain, improving binary classification accuracy by 1.51–2.02% and eight-class accuracy by 1.84–2.94%, confirming the complementary effectiveness of global and spatial-frequency feature fusion.

#### 5.5.2 BACH dataset.

Table 11 presents the four-class classification results of the Baseline, ModelA, ModelB, and ModelC models on the BACH dataset. From Table 11, baseline model attains 97.26% accuracy; ModelA effectively introduces the wavelet low-frequency image and statistical transformer module, improving the classification accuracy to 98.93% (relative improvement+1.67%); ModelB introduces the Fourier high-frequency components together with the DGFEM module, enhancing the spatial characterization and yielding an accuracy of 98.21% (a 0.95% improvement). ModelC combines the advantages of ModelA and ModelB to further improve the classification ability of ModelC, and the classification result reaches 99.35%.

**Table 11:** Classification performance of ablation experiments on BACH dataset.

Model	Acc (%)	Pre (%)	Rec (%)	F1-Sc (%)
Baseline	97.26	97.19	97.27	97.16
ModelA	98.93	98.78	98.97	98.87
ModelB	98.21	98.01	98.22	98.10
ModelC	99.35	99.37	99.24	99.30

The experimental results fully verify the effectiveness and synergy of each module. Especially, while maintaining high accuracy, ModelC has achieved more than 99% of the evaluation indicators, showing the stability and reliability of classification.

### 5.6 Comparison with classic models

#### 5.6.1 BreakHis dataset.

**Fig 8:**
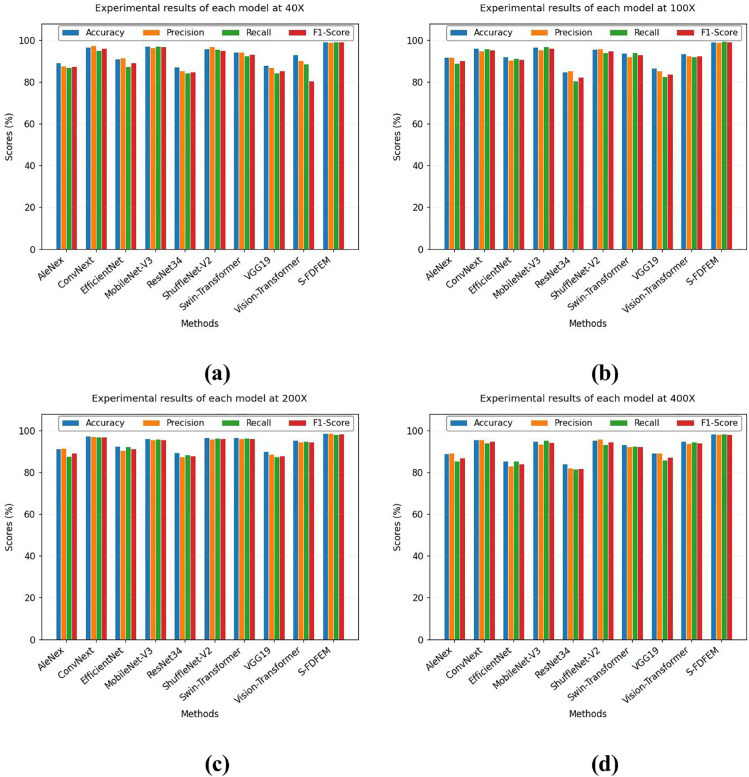
Visualization of experimental results of binary classification on BreakHis Dataset.

The existing classic networks AleNex [40], ConvNext-V2 [41], EfficientNet-V2 [42], MobileNet-V3 [43], ShuffleNet-V2 [44], Vision-Transformer [45], ResNet34 [37], VGG19 [46], and Swin-Transformer [47] are chosen to comprehensively verify the performance of the proposed S-FDFEM. Tables 12 and 13 show the compared results of binary classification and 8-class classification on BreakHis Dataset. Fig 8 shows the visualization of indicators of binary classification. Table 12 presents the classification accuracies of the MobileNet-V3 model on the BreakHis dataset, achieving 97.32%, 96.63%, 96.35%, and 95.05% at 40×, 100×, 200× and 400× magnifications, respectively. The S-FDFEM model performs well and can accurately capture the features of complex pathological images. Its accuracy at four multiples is 2.01%, 2.73%, 2.32%, and 3.48% higher than that of the Mobilenet-V3 network. From Table 13, though the indicators of all models are low, the S-FDFEM also shows the highest indicators. Compared with mobilenet-v3, the S-FDFEM improves accuracy by 0.37-0.96%, precision by 0.39-2.08%, Recall by 0.58-2%, and the F1-score by 1.19-1.79%.

**Table 12:** Compared results in the binary classification on BreakHis Dataset.

Model	40×				100×			
	Acc (%)	Pre (%)	Rec (%)	F1-Sc (%)	Acc (%)	Pre (%)	Rec (%)	F1-Sc (%)
AleNex	89.30	87.80	87.11	87.44	91.98	91.93	89.05	90.30
ConvNext-V2	96.82	97.42	95.21	96.21	96.15	95.07	96.07	95.54
EfficientNet-V2	91.14	91.54	87.57	89.20	92.15	90.52	91.31	90.90
MobileNet-V3	97.32	96.53	97.32	96.91	96.63	95.40	96.99	96.13
ResNet34	87.30	85.57	84.49	84.99	84.90	85.36	80.64	82.41
ShuffleNet-V2	95.99	97.04	95.73	95.17	95.83	95.92	94.27	95.03
Swin-Transformer	94.48	94.41	92.63	93.45	93.91	92.09	94.30	93.06
VGG19	87.96	86.88	84.54	85.55	86.70	85.45	82.65	83.83
Vision-Transformer	93.14	90.36	88.74	80.49	93.75	92.54	92.00	92.72
**Proposed**	**99.33**	**99.08**	**99.37**	**99.22**	**99.36**	**98.98**	**99.54**	**99.25**
**Model**	**200×**				**400×**			
	**Acc (%)**	**Pre (%)**	**Rec (%)**	**F1-Sc (%)**	**Acc (%)**	**Pre (%)**	**Rec (%)**	**F1-Sc (%)**
AleNex	91.37	91.71	87.81	89.43	89.17	89.37	85.47	87.02
ConvNext-V2	97.51	97.15	97.01	97.08	95.60	95.81	94.07	94.87
EfficientNet-V2	92.54	90.67	92.37	91.44	85.50	83.15	85.58	84.06
MobileNet-V3	96.35	95.60	95.87	95.74	95.05	93.65	95.45	94.45
ResNet34	89.55	87.41	88.58	87.95	84.22	82.10	81.51	81.79
ShuffleNet-V2	96.68	95.87	96.41	96.14	95.41	96.02	93.49	94.61
Swin-Transformer	96.85	96.12	96.53	96.32	93.39	92.34	92.60	92.47
VGG19	90.05	88.91	87.44	88.12	89.36	89.19	86.05	87.35
Vision-Transformer	95.52	94.58	94.98	94.78	94.86	93.81	94.57	94.18
**Proposed**	**98.67**	**98.74**	**98.15**	**98.44**	**98.53**	**98.19**	**98.47**	**98.33**

**Table 13:** Compared results in eight-class classification on BreakHis Dataset.

Model	40×				100×			
	Acc (%)	Pre (%)	Rec (%)	F1-Sc (%)	Acc (%)	Pre (%)	Rec (%)	F1-Sc (%)
AleNex	87.71	87.17	84.15	85.30	87.92	85.87	88.91	86.85
ConvNext-V2	92.59	92.45	90.65	91.44	88.08	88.85	85.72	86.45
EfficientNet-V2	80.81	79.86	78.49	78.99	78.10	74.25	75.91	74.79
MobileNet-V3	93.60	93.04	92.08	92.44	91.95	91.71	90.57	91.00
ResNet34	72.56	70.75	63.82	66.49	65.38	59.29	55.65	55.14
ShuffleNet-V2	91.92	91.23	90.35	90.57	88.89	87.41	88.98	88.14
Swin-Transformer	81.48	79.42	76.20	77.59	82.28	82.58	76.13	78.71
VGG19	87.21	85.74	85.35	85.40	87.76	87.07	86.35	86.60
Vision-Transformer	51.75	24.23	26.77	24.22	56.97	43.20	33.26	32.25
**Proposed**	**94.11**	**93.43**	**93.96**	**93.64**	**92.91**	**93.16**	**92.57**	**92.79**
**Model**	**200×**				**400×**			
	**Acc (%)**	**Pre (%)**	**Rec (%)**	**F1-Sc (%)**	**Acc (%)**	**Pre (%)**	**Rec (%)**	**F1-Sc (%)**
AleNex	85.33	83.65	81.33	81.79	84.35	82.24	82.05	83.46
ConvNext-V2	88.67	85.75	86.63	85.51	86.00	86.41	80.73	83.06
EfficientNet-V2	79.93	81.16	72.49	75.56	74.17	71.57	65.84	68.00
MobileNet-V3	90.67	89.41	87.95	88.51	89.50	87.91	86.96	87.35
ResNet34	65.67	58.62	50.05	52.72	63.53	58.85	45.21	46.85
ShuffleNet-V2	89.17	87.23	86.10	86.47	84.32	84.29	79.81	81.82
Swin-Transformer	80.66	76.99	73.45	74.41	80.11	75.86	73.55	74.28
VGG19	53.83	34.68	31.06	31.81	54.33	34.87	31.37	31.99
Vision-Transformer	60.54	51.06	36.56	35.88	60.71	58.08	39.81	41.06
**Proposed**	**91.17**	**91.02**	**88.55**	**89.70**	**89.87**	**89.99**	**87.54**	**88.71**

**Fig 9:**
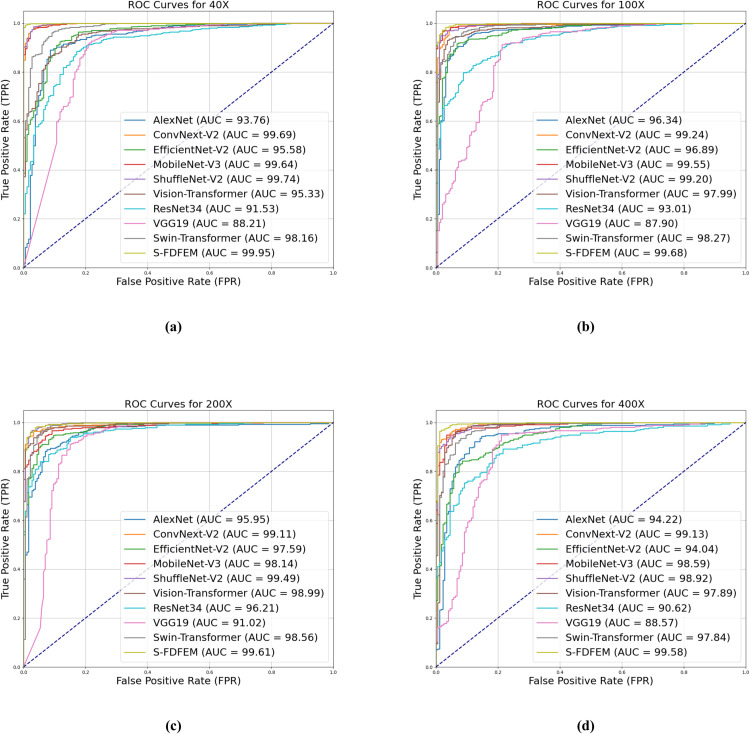
ROC curves of the experimental results of binary classification on BreakHis Dataset.

Fig 9 shows the ROC curves of Classic models and S-FDFEM on the BreakHis Dataset. The proposed S-FDFEM achieves AUC Value of 99.95 (40×), 99.68 (100×), 99.61 (200×), and 99.58 (400×), consistently exceeding Classic models across all magnification levels, which demonstrates S-FDFEM’s stable discriminative ability in distinguishing histopathological samples.

#### 5.6.2 Comparison on bach dataset.

**Fig 10:**
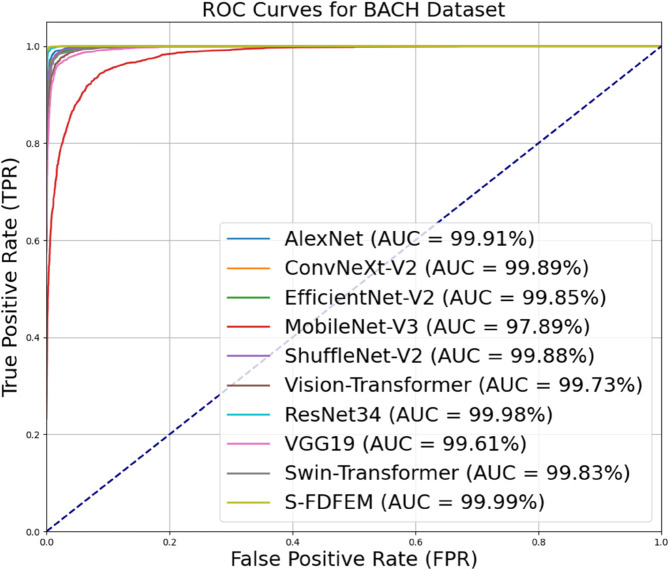
ROC curves of experimental results of four-class classification on BACH Dataset.

Table 14 shows the comparative results between S-FDFEM and classical networks shown in the Sect 5.6.1 on BACH Dataset, and Fig 10 illustrates their corresponding ROC curves. From Table 14, S-FDFEM achieves the best overall performance, with ResNet34 ranking second.Though the accuracy difference is small, S-FDFEM shows more comprehensive performance advantages: accuracy (99.35%), precision (99.37%), recall (99.24%) and F1 score (99.30%). These four indicators have exceeded 99% and tend to be stable. From Fig 10, the AUC value of S-FDFEM reaches 99.99, which is significantly better than other models. These results verify the effectiveness of the space-frequency domain collaborative design in enhancing classification robustness and reducing the risk of misdiagnosis.

**Table 14:** Compared results on BACH Dataset.

Model	Acc (%)	Pre (%)	Rec (%)	F1-Sc (%)
AlexNet	98.48	98.25	98.40	98.33
ConvNext-V2	98.52	97.89	98.76	98.28
EfficientNet-V2	98.63	98.37	98.74	98.55
MobileNet-V3	87.70	86.61	86.61	86.60
ResNet34	98.97	98.83	99.09	98.95
ShuffleNet-V2	97.18	96.81	97.25	97.02
Swin-Transformer	96.73	96.37	96.69	96.52
VGG19	95.05	94.78	94.55	94.66
Vision-Transformer	95.58	95.01	95.72	95.29
**S-FDFEM**	**99.35**	**99.37**	**99.24**	**99.30**

### 5.7 Comparison with existing models

We choose the existing models DsHoNet [23], MAW-BMRSFAN [25], DenseNet201 + XGBoost [48], Multi-Level Feature Fusion [49], PCSAM- ResCBAM [50], MSFEL-DAAMS [51], GLNET [52] and FCCS-Net [53] on binary classification to be compared with the S-FDFEM. Due to the serious data imbalance problem in the images of eight categories in the BreakHis dataset, which seriously affects the classification results, this paper did not correct the data imbalance. To ensure the effectiveness and fairness of the comparison, we selected four models with the same untreated data imbalance problem for comparison: ResNet-FRLM [54], IDSNe [55], SE-ResNet [56] and AFFNet [57].

From Table 15, the proposed S-FDFEM exhibits significant classification advantages at 40×, 100×, and 400× magnifications, whose accuracies increase 1.55%, 0.96%, and 0.79% higher than those of the suboptimal model DsHoNet. Only at 200× magnification, the optimal model DsHoNet has a slight advantage of 0.34% on accuracy over the suboptimal model S-FDFEM. Therefore, S-FDFEM model outperforms other models and has good performances.

**Table 15:** Compared accuracy of binary classification task on BreakHis Dataset.

Methodology	40×	100×	200×	400×
DsHoNet	97.78	98.40	99.01	97.74
MAW-BMRSFAN	85.69	85.08	86.38	85.13
DenseNet201 + XGBoost	93.60	91.30	93.80	89.10
Multi-Level Feature Fusion	95.20	95.80	95.60	95.10
PCSAM-ResCBAM	95.14	97.09	98.74	97.99
MSFEL-DAAMS	95.42	93.79	94.87	97.08
GLNET	90.01	92.32	91.98	91.51
FCCS-Net	96.79	95.12	96.72	94.45
**S-FDFEM**	**99.33**	**99.36**	**98.67**	**98.53**

From Table 16, S-FDFEM exhibits the highest classification performance at 40×magnification, surpassing SE-ResNet by 0.37%. The best SE- ResNet performs slightly better than S-FDFEM at magnifications of 100×, 200× and 400×, with the difference remaining within 1%.These results indicate that the S-FDFEM method maintains strong competitiveness and has strong overall robustness.

**Table 16:** Compared accuracy of 8-class classification task on BreakHis Dataset.

Methodology	40×	100×	200×	400×
ResNet-FRLM	91.21	89.94	87.65	83.44
IDSNe	89.10	85.00	87.00	84.50
SE-ResNet	93.74	93.81	92.22	90.66
AFFNe	93.00	90.62	90.32	90.65
**S-FDFEM**	**94.11**	**92.91**	**91.17**	**89.87**

In summary, the proposed S-FDFEM demonstrates consistent and robust performance in both binary and multi-class breast cancer classification tasks, maintaining a competitive advantage over most existing approaches. This confirms its effectiveness and adaptability in analyzing breast cancer histopathological images.

To sum up, the S-FDFEM shows the robust performance on both binary classification and 8-class classification and still maintains its competitive advantage over most existing methods, highlighting its effectiveness and adaptability in processing breast cancer pathological images.

### 5.8 The impact of different loss functions

We conducted comparative experiments on the BreakHis dataset for the eight-class classification task using both Cross-entropy loss and Focal loss. As shown in Table 17, the conventional cross-entropy loss leads to a noticeable decline in classification performance under imbalanced data, as it tends to bias the model toward majority classes. In contrast, the focal loss alleviates this issue by adaptively down-weighting easily classified samples, thereby guiding the model to focus more on minority and hard-to-classify categories. Consequently, the focal loss enables the model to learn more discriminative feature representations and improves overall classification robustness.

**Table 17:** Compared results of 8-class classification task on BreakHis Dataset.

Loss Function	40×				100×			
	Accuracy (%)	Precision (%)	Recall (%)	F1-Score (%)	Accuracy (%)	Precision (%)	Recall (%)	F1-Score (%)
Cross Entropy	88.46	87.32	85.34	86.22	87.02	87.15	88.91	86.85
Focal Loss	94.11	93.43	93.96	93.64	92.91	93.16	92.57	92.79
**Loss Function**	**200×**				**400×**			
	**Accuracy (%)**	**Precision (%)**	**Recall (%)**	**F1-Score (%)**	**Accuracy (%)**	**Precision (%)**	**Recall (%)**	**F1-Score (%)**
Cross Entropy	84.91	82.29	82.39	82.34	82.57	81.55	81.18	80.49
Focal Loss	91.17	91.02	88.55	89.70	89.87	89.99	87.54	88.71

## 6 Visualization

**Fig 11:**
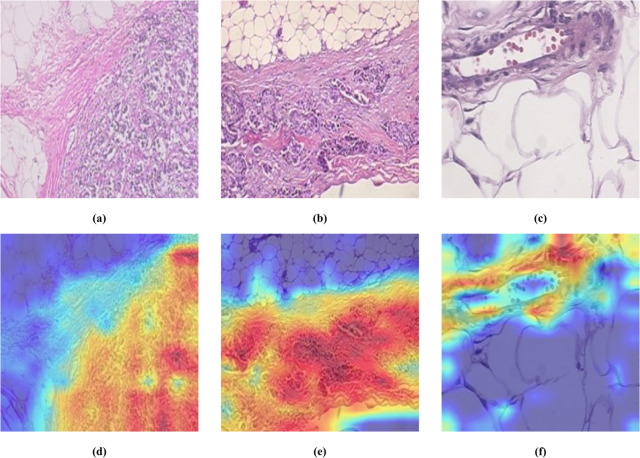
Heatmap Visualization Analysis of Pathological Images of Breast Cancer in BreakHis Datasets.

We employed Grad-CAM visualization to analyze the BreakHis datasets, shown in Fig 11. Fig 11 illustrates the model’s attentional focus on pathological images from the BreakHis dataset. The results indicate that the S-FDFEM model can accurately locate pathological areas with irregular shapes, which is due to the model’s recognition of the spatial distribution of cells and tissues and adaptive learning of irregular contours. This performance highlights the robustness of the model in capturing fine-grained spatial features, which is crucial for improving the diagnostic accuracy of histopathological analysis of tissues.

## 7 Conclusion

The proposed S-FDFEM integrates spatial and frequency domain information to improve the automated classification of breast cancer histopathological images. In the spatial domain, deformable bottleneck convolution extracts irregular local features from pathological images. In the frequency domain, wavelet and Fourier transforms capture global and spatial characteristics: Wavelet low-frequency components combined with a Statistical Transformer, establishing long-range dependencies and extracting key pathological features. Fourier high-frequency components paired with a deep gradient feature extraction module encode spatial relationships, enhancing discriminative feature representation. By fusing these complementary features, S-FDFEM generates richer and more distinctive deep representations. Evaluated on the BreakHis Dataset at 40×, 100×, 200× and 400× magnifications, S-FDFEM achieves the accuracies 99.33%, 99.36%, 98.64%, and 98.53% on binary classification, and the accuracies of 94.11%, 92.91%, 91.17%, and 89.87% on 8-class classification, respectively. Additionally, it attains 99.35% accuracy on the BACH Dataset. These results demonstrate the robustness of S-FDFEM, highlighting the potential of hybrid spatial-frequency approaches to enhance breast cancer pathological image classification. In the future, we will conduct research on the unresolved issue of data imbalance in this article, reduce the impact of data distribution, fully utilize the potential of the model, and further improve classification performance.
